# The NLRP3 inhibitor Dapansutrile improves the therapeutic action of lonafarnib on progeroid mice

**DOI:** 10.1111/acel.14272

**Published:** 2024-08-27

**Authors:** Inés Muela‐Zarzuela, Juan Miguel Suarez‐Rivero, Daniel Boy‐Ruiz, Juan López‐Pérez, Marta Sotelo‐Montoro, Maria del Mar Navarrete‐Alonso, Isidro G. Collado, José Manuel Botubol‐Ares, Alberto Sanz, Mario D. Cordero

**Affiliations:** ^1^ Department of Molecular Biology and Biochemical Engineering Universidad Pablo de Olavide Seville Spain; ^2^ Department of Immunology Puerta del Mar Hospital Cádiz Spain; ^3^ Instituto de Investigación e Innovación Biomédica de Cádiz, INiBICA Hospital Universitario Puerta del Mar Cádiz Spain; ^4^ Departamento de Química Orgánica, Facultad de Ciencias, Campus Universitario Río San Pedro s/n, Torre Sur, 4a Planta University of Cádiz Cádiz Spain; ^5^ School of Molecular Biosciences, College of Medical, Veterinary and Life Sciences University of Glasgow Glasgow UK

**Keywords:** Dapansutrile, Lonafarnib, NLRP3, progeria

## Abstract

The role of the inflammasomes in aging and progeroid syndromes remain understudied. Recently, MCC950, a NLRP3 inhibitor, was used in Zmpste24^−/−^ mice to ameliorate the phenotypes. However, the safety of MCC950 was questioned due to liver toxicity observed in humans. Nevertheless, inhibition of the inflammasomes would be a beneficial therapy for progeria. Here, we show that OLT1177 (dapansutrile), other NLRP3 inhibitor, improved cellular and animal phenotypes using progeroid fibroblasts and a Lmna^G609G/G609G^ mouse model. In both cases dapansutrile reduced progerin accumulation, NLRP3‐inflammasome activation and secretory phenotype of senescence, extended the lifespan of progeroid animals, preserved bodyweight, and reduced kyphosis, inflammation, and senescence. Interestingly, dapansutrile further improved the effect of lonafarnib, the only FDA‐approved drug for the progeria. The combination of both drugs reduced the inflammation and senescence, extended survival and ameliorated various progeroid defects both in vitro and in vivo, compared with treatment using lonafarnib alone. These findings and the safety of dapansutrile demonstrated in several clinical trials proposes it as a possible co‐adjuvant treatment with lonafarnid in HGPS.

## Graphical abstract

The combination therapy of lonafarnib and dapansutrile, and NLRP3 inhibitor show beneficial effect improving lifespan and healthspan by inhibition of inflammation.

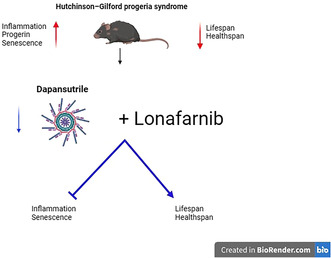

## 1 INTRODUCTION

Hutchinson‐Gilford progeria syndrome (HGPS) is a rare premature aging disease in which a point mutation in the LMNA gene causes the accumulation of aberrant lamin A at the nuclear envelope. This results in the disruption of the nuclear membrane architecture with progerin protein accumulation, and abnormal gene transcription and signal transduction (Schreiber & Kennedy, 2013). The clinical phenotype is marked by an important physiological and physical decline (Schreiber & Kennedy, 2013). Inflammation has been associated to the pathophysiology of HGPS. For instance, the Nuclear factor κB (NF‐κB) mediates the secretion of high levels of proinflammatory cytokines, including interleukin 6 (IL‐6) in the mouse HGPS models Lmna^G609G/G609G^ and Zmpste24^−/−^. The last model is associated with the proteolytic processing of farnesylated prelamin A, the precursor of the nuclear scaffold protein lamin A (Osorio et al., 2011, 2012; Squarzoni et al., 2021). Interestingly, pharmacological treatment with anti‐inflammatories prevent age‐associated features in these models, improve many of the symptoms and extends the longevity of mouse models (Osorio et al., 2012; Squarzoni et al., 2021).

Recently, our group described the role of the NLRP3‐inflammasome, a well‐known multiprotein complex important for the maturation of IL‐1β and IL‐18, on the pathophysiology of HGPS both in human fibroblasts from patients and Zmpste24^−/−^ mice. Our findings showed that the specific inhibition of NLRP3 by MCC950 improved cellular features associated with progeria, and more important, extended the lifespan of progeroid animals (González‐Dominguez et al., 2021). However, the pharmacological development of MCC950 is currently uncertain due to an increased risk of liver toxicity observed after phase 1b testing (Coss, 2020). So, safer treatment options for HGPS patients are urgently required to reduce activation of the NLRP3 inflammasome and prevent damaging inflammation.

Dapansutrile (OLT1177) is a new small molecule that specifically targets the NLRP3 inflammasome and prevents the activation of caspase‐1 and the maturation and release of IL‐1β (Marchetti et al., 2018). A phase 1 trial has shown that dapansutrile is safe in humans (Marchetti et al., 2018) and the lead is currently undergoing Phase 2 clinical studies for the treatment of several inflammatory conditions, such as osteoarthritis, inflammatory, and cardiovascular diseases, among others (Klück et al., 2020; Marchetti et al., 2018; Wohlford et al., 2020). According to this, we investigate for the first time the effects of dapansutrile in skin fibroblasts from patients with the classical mutation (LMNA c.1824C>T) and its use as an acute, oral pharmacological intervention in Lmna^G609G/G609G^ mice. Dapansutrile not only reduced progerin levels and inhibited inflammation and senescence, but it also improved longevity and ameliorated the pathopysiological phenotype.

## 2 MATERIALS AND METHODS

### 2.1 Reagents

Trypsin was purchased from Sigma Chemical Co., (St. Louis, Missouri). Anti‐actin monoclonal antibody from Calbiochem‐Merck Chemicals Ltd. (Nottingham, UK). Progerin, p16, p21, p53, NLRP3, NLRP1, IL‐1β, and caspase 1 were obtained from Cell Signaling Technology. NLRP3 inhibitors OLT1177 (dapansutrile) were obtained from Sigma‐Aldrich (Saint Louis, USA). A cocktail of protease inhibitors (complete cocktail) was purchased from Boehringer Mannheim (Indianapolis, IN). Grace's insect medium was purchased from Gibco. The Immun Star HRP substrate kit was from Bio‐Rad Laboratories Inc. (Hercules, CA).

### 2.2 Fibroblast culture

All fibroblasts from patients with HGPS were obtained from The Progeria Research Foundation Cell and Tissue Bank (http://www.progeriaresearch.org). The following fibroblasts were used: HGADFN367 (3‐year‐old male) and HGADFN155 (1.2‐year‐old female). Control fibroblasts was used the HGADFN368 (37‐year‐old female), mother of the HGADFN367. Fibroblasts were cultured in high glucose DMEM (Dulbecco's modified media) (Gibco, Invitrogen, Eugene, OR, USA) supplemented with 15% fetal bovine serum (FBS) (Gibco, Invitrogen, Eugene, OR, USA), 1% GlutaMAX (ThermoFisher) and antibiotics (Sigma Chemical Co., St. Louis, MO, USA). Cells were incubated at 37°C in a 5% CO_2_ atmosphere. The medium was changed every 2 days to avoid changes in pH.

THP‐1 cells were seeded in six‐wells plates in RPMI medium supplemented with 10% FBS and 1% antibiotics (Thermo Fisher, Waltham, MA, USA, 11548876) until 90% confluence. Then, THP1 cells were differentiated into macrophages using phorbol‐12‐myristate‐13‐acetate (PMA) at 50 nM for 24 h. For patient's serum assay, THP1‐derived macrophages medium was refreshed with RPMI without FBS and supplemented with Conditioned Medium (CM).

### 2.3 Conditioned medium

Healthy an HGPS fibroblasts were seeded in a 10 cm dish and incubated for 2 days in DMEM with 0.5% FBS. After incubation, the conditioned medium (CM) was collected, centrifuged at 5000 g and filtered through a 0:2 μm pore filter. CM was mixed with DMEM 40% FBS in a proportion of 3 to 1 to generate CM containing 10% FBS.

### 2.4 Immunofluorescence assay

Fibroblasts were grown on 1 mm width glass coverslips for 72 h in high glucose DMEM medium containing 10% FBS and 1% antibiotics. They were washed twice with PBS, fixed in 3.8% paraformaldehyde for 15′ at room temperature, permeabilized with 0.1% Triton X‐100 in PBS for 10′ and incubated in blocking buffer (BSA 1%, Triton X‐100 0.05% in PBS) for 30′. In the meantime, the primary antibody was diluted 1:100 in antibody buffer (BSA 0.5%, Triton X‐100 0.05% in PBS). Fibroblasts were incubated overnight at 4°C with the primary antibody and subsequently washed twice with PBS. The secondary antibody was similarly diluted 1:400 in antibody buffer, but their incubation time on cells was reduced to 2 h at room temperature. Coverslips were then washed twice with PBS, incubated for 5′ with PBS containing DAPI 1 μg/mL and washed again with PBS. Next, they were mounted on microscope slides using Vectashield Mounting Medium (Vector Laboratories, Burlingame, CA, USA, H1000).

### 2.5 Real‐time quantitative PCR (qPCR)

Expression of NLRP1, NLRP3, ASC, and caspase 1 was analysed by SYBR Green (Takara, Kusatsu, Japan, RR420W) quantitative PCR. cDNA was obtained from extracted mRNA using the iScript cDNA KIT (Biorad, Hercules, CA, USA). The qPCR was performed in a CFX96 Connect Real‐Time PCR Detection System (Biorad, Hercules, CA, USA). Ribosomal 18S expression was used as reference gene. 2^−^ΔΔCT method was used as a relative quantification strategy for data analysis. This method is a convenient way to calculate relative gene expression levels between different samples in that it directly uses the threshold cycles (CTs) generated by the qPCR system for calculation. Every primer's sequence could be checked in Table S1.

### 2.6 Western blotting

Whole cellular lysate from fibroblasts was prepared by gentle shaking with a buffer containing 0.9% NaCl, 20 mM Tris‐ClH, pH 7.6, 0.1% Triton X‐100, 1 mM phenylmethylsulfonylfluoride and 0.01% leupeptin. The protein content was determined by the Bradford method. Electrophoresis was carried out in a 10%–15% acrylamide SDS/PAGE and proteins were transferred to Immobilon membranes (Amersham Pharmacia, Piscataway, NJ). Next, membranes were washed with PBS, blocked over night at 4°C and incubated with the respective primary antibody solution (1:1000). Membranes were then probed with their respective secondary antibody (1:2500). Immunolabeled proteins were detected by chemiluminescence method (Immun Star HRP substrate kit, Bio‐Rad Laboratories Inc., Hercules, CA). Western blot images were quantified using ImageJ software.

### 2.7 Nuclear morphology

For nuclear deformation cell quantification, nuclei were stained with DAPI. After staining, abnormal nuclei were counted in randomly selected fields (minimum 100 cells per case) and expressed as percentages of total cells counted. Counting of cells with nuclear deformation was performed by three independent observers.

### 2.8 Proliferation assay

Fibroblasts were seeded in 12‐well plates. One hundred thousand fibroblasts were cultured with or without the dapansutrile, lonafarnib, or combination for 24, 48, and 120 h. To measure proliferation rate, fibroblasts was seeded and after treatment, cells were harvested and quantified by using a TC10™ Automated Cell Counter (Bio‐Rad). Cell viability was assessed by trypan blue exclusion. Proliferation rate was calculated by total cell number. Results are expressed as mean ± SD of two independent assays.

### 2.9 ELISA (enzyme‐linked immunosorbent assay)

IL‐6, IL‐1β, and IL‐18 levels were assayed in supernatant by duplicate using commercial ELISA kits (Thermo Fisher Scientific, MA, USA).

### 2.10 Cytokine array

Blood serum was collected from wild‐type and Lmna^G609G/G609G^ mice. In an in vitro model healthy and HGPS fibroblasts were cultured in serum‐free media for 24 h and media were collected for analysis. Media and blood serum were analyzed for expression of several mouse cytokine and chemokines (MD44) or human cytokine and chemokines (HD48), respectively, using a Multiplexing LASER Bead Assay (Eve Technologies).

### 2.11 Animals

Animal studies were performed in accordance with European Union guidelines (2010/63/EU) and the corresponding Spanish regulations for the use of laboratory animals in chronic experiments (RD 53/2013 on the care of experimental animals). All experiments were approved by the local institutional animal care committee. For all experiments, only male mice were used. Mutant mice Lmna^G609G/G609G^ have been described previously (Osorio et al., 2011). All groups had ad libitum access to their prescribed diet and water throughout the whole study. Body weight was monitored weekly. Animal rooms were maintained at 20°C–22°C with 30%–70% relative humidity.

For all experiments with NLRP3 inhibitors, wild type and Lmna^G609G/G609G^ were maintained on a regular 12 h light/dark cycle at 20°C–22°C. Treatments were started at 1 month of age after randomization into four groups (wild type vehicle, Lmna^G609G/G609G^, Lmna^G609G/G609G^ dapansutrile, Lmna^G609G/G609G^ lonafarnib, and Lmna^G609G/G609G^ dapansutrile+Lonafarnib). The mice received dapansutrile (60 mg/kg) via drinking water for the survival period and lonafarnib, provided by The Progeria Research Foundation Cell and Tissue Bank, was given daily at a dose of 450 mg per kg of soft gel‐based chow (a dose recommended by the Foundation).

All groups had ad libitum access to their prescribed diet and water throughout the study. Individuals were monitored daily and weighed monthly but were otherwise left undisturbed until they died. Survival was assessed using male mice, and all animals were dead by the time of this report. Kaplan–Meier survival curves were constructed using known birth and death dates, and differences between groups were evaluated using the logrank test.

### 2.12 Statistical analysis

Data in the figures are shown as mean ± SD. Data between different groups were analysed statistically by using ANOVA on Ranks with Sigma Plot and Sigma Stat statistical software (SPSS for Windows, 19, 2010, SPSS Inc. Chicago, IL, USA). For cell‐culture studies, Student's *t* test was used for data analyses. A value of *p* < 0.05 was considered significant.

## 3 RESULTS

### 3.1 Dapansutrile reduces inflammasome expression in LMNA c.1824C>T patient fibroblasts

**FIGURE 1:**
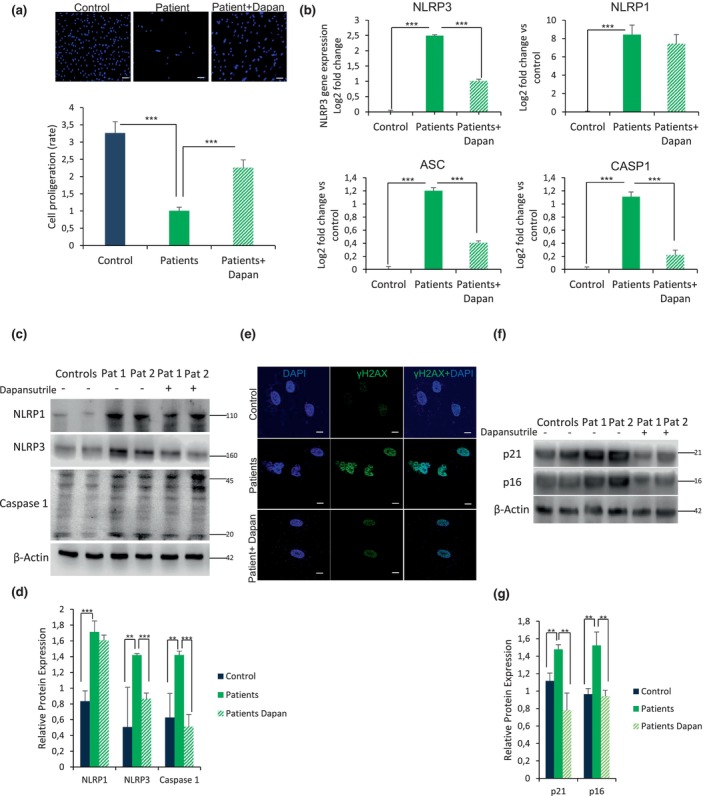
Dapansutrile reduces skin fibroblasts inflammasome activation, senescence and progeroid phenotypes. (a) Cell growth with Dapansutrile determined in healthy and HGPS fibroblasts from eight patients. (b) Effect of Dapansutrile on mRNA expression of NLRP1, NLRP3, ASC and Caspase 1 determined by qPCR experiments in skin fibroblasts, *n* = 3 (controls) and *n* = 8 (patients). (c, d) Western blot analysis showing representative blots of NLRP1, NLRP3, caspase 1, and β‐Actin levels in skin fibroblasts from patients with HGPS, *n* = 2 (controls) and *n* = 2 (patients). (e) Immunofluorescence (IF) visualization of γH2AX (green) and nuclei (blue) in skin fibroblasts from a representative patient and control. (f, g) Protein expression of p21 and p16 in skin fibroblasts from patients with HGPS, *n* = 4 (controls) and *n* = 4 (patients) (Two more patients and control in Figure S1). Data are shown as means ± SD. ***p < 0.001, **p < 0.005.

To evaluate the phenotype associated to aging in HGPS fibroblasts, all our experiments used control and patient fibroblasts at passage P7. To investigate the cytotoxic effects of dapansutrile on HGPS fibroblasts, we assessed cell viability using the TC10™ Automated Cell Counter. As shown in Figure 1a, a low dose of dapansutrile (1 μM) not only exhibited no obvious cytotoxicity on patient fibroblasts after 72 h, but also showed a statistically significant dose‐dependent increase in the growth rate of patient fibroblasts (Figure 1a).

To evaluate the role of the NLRP3 inflammasome in patients with the classical mutation (LMNA c.1824C>T), we analyzed the expression levels of several genes that are part of the inflammasome complex, including NLRP3, NLRP1, caspase‐1, and. We found a significant increase in gene expression for NLRP3, NLRP1, ASC, and Caspase 1 in HGPS fibroblasts compared with controls (Figure 1b). Interestingly, dapansutrile caused a significant inhibition of NLRP3, caspase 1, and ASC gene expression without changing the levels of NLRP1.

Immunoblotting analysis corroborated higher protein expression levels of NLRP3, NLRP1, and caspase 1 in HGPS fibroblasts (Figure 1c and Figure S1). To confirm the increase in inflammation, we measured the level of the pro‐inflammatory cytokines IL‐6, IL‐1β, and IL‐18 in the cellular supernatant. HGPS cells treated with 1 μM dapansutrile showed a significantly reduced release of IL‐1β and IL‐6 but not change in IL‐18 compared to control cells (Figure S2), supporting the hypothesis that dapansutrile leads to a partial rescue of a general pro‐inflammatory response at the cellular level.

### 3.2 Dapansutrile reduces nuclear deformation and reduces senescence markes in HGPS fibroblasts

A morphological characteristic of HGPS cells is the irregularly shaped nuclei that display blebs, folds, and herniations in the nuclear envelope associated with the accumulation of truncated protein progerin (Capell et al., 2005; Goldman et al., 2004). When we evaluated the effect of dapansutrile treatment on the nuclear morphology of HGPS fibroblasts, we observed the reduction in the frequency of abnormal nuclear morphology in both control and HGPS fibroblasts (Figure 1d and Figure S3a). Furthermore, altered nuclear morphology is usually associated with senescence as previously shown (Mojiri et al., 2021). Accordingly, γH2XA levels were increase in HGPS nuclei, returning to basal levels following dapansutrile treatment (Figure 1d and Figure S3b). Besides, dapansutrile induced a significant reduction in gene (Figure S4) and protein expression of two other two senescence markers such as p16 and p21 (Figure 1e and Figure S1).

### 3.3 Dapansutrile improves inflammation and survival in Lmna^G609G^ ^/G609G^ mice

**FIGURE 2:**
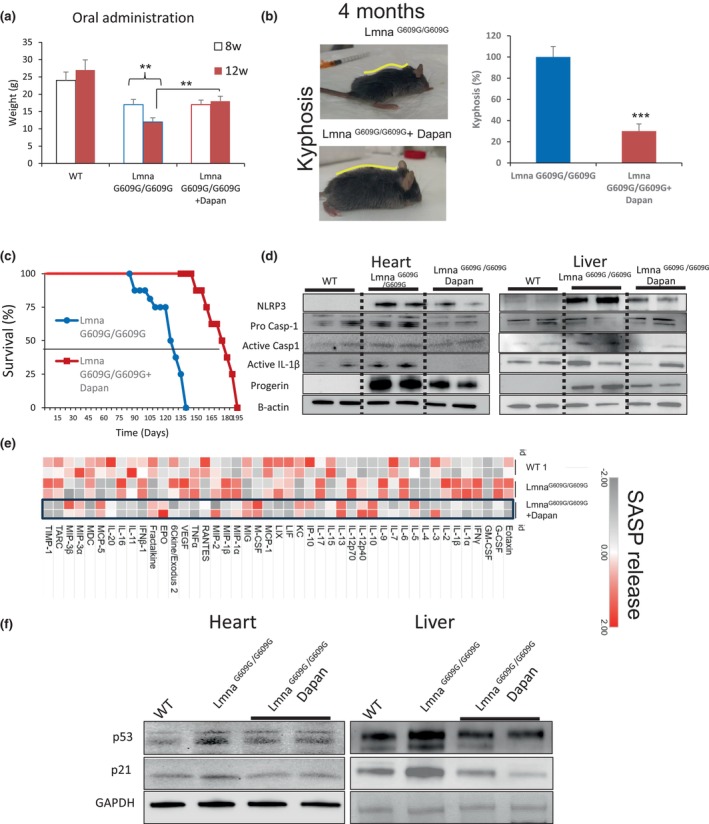
NLRP3 Inhibition by dapansutrile ameliorates Lmna^G609G/G609G^ mice progeroid phenotypes. (a) Body weights of the indicated groups at 8 and 12 weeks after treatments. (b) Kyphosis percentage of untreated (*n* = 6 males) and dapansutrile‐treated (*n* = 6 males) Lmna^G609G/G609G^ mice. Image show two representative animal after 4 months of treatment. (c) Kaplan–Meier graph showing a significant increase in the medium and maximum lifespan in treated mice compared with Lmna^G609G/G609G^ mice. *N* = 8 per group. (d) Western blot analysis showing representative blots of NLRP3, total and active caspase 1, active IL‐1β, progerin and β‐Actin levels in heart and liver tissues from wild‐type, Lmna^G609G/G609G^ and dapansutrile‐treated Lmna^G609G/G609G^ mice. *n* = 5 mice per group. Densitometry in Figure S5. (e) A heat map depicting the expression of 44 mouse cytokines in the serum of WT, Lmna^G609G/G609G^, and dapansutrile‐treated Lmna^G609G/G609G^ mice. (f) Western blot analysis showing representative blots of p53, p21 and β‐Actin levels in heart and liver from wild‐type, Lmna^G609G/G609G^ and dapansutrile‐treated Lmna^G609G/G609G^ mice. Densitometry in Figure S6. *n* = 5 mice per group. ***p < 0.001, **p < 0.005.

To extend our in vitro observations to an in vivo model, we evaluated the effect of dapansutrile using progeroid mice. We explored two different forms for the treatment of dapansutrile; intraperinoteal (i.p.) injection three times per week vs oral administration on the water drinking. Only oral administration preserved the body weight loss (Figure 2a and Figure S5). Moreover, we found that the percentage of animal with kyphosis was reduced in oral dapansutrile‐treated mice (Figure 2b), and the treatment notably extended the survival of Lmna^G609G/G609G^ mice by 40% of mean and 39% of maximun (Figure 2c). Furthermore, as observed in cultured HGPS cells, in vivo dapansutrile administration reduced NLRP3, active caspase 1 and active IL‐β protein expression and progerin accumulation in Lmna^G609G/G609G^ heart and liver tissues and interestingly the downstream of gasdermin D cleavage which is usually associated to pyroptotic cell death (Figure 2d and Figure S6).

Finally, to illustrate the effect of dapansutrile on senescence in vivo, we measured the expression of the most prominent SASP members in the serum of Lmna^G609G/G609G^ treated mice, utilizing a cytokine array. Lmna^G609G/G609G^ mice showed increased serum levels of various inflammatory factors, including SASP. The response was largely abrogated by dapansutrile treatment (Figure 2e). Dapansutrile also induced a significant reduction in the levels of p21 and p53 in heart and liver (Figure 2f and Figure S7). These results suggest that the inhibition of NLRP3 by dapansutrile reduces both the release of SASP factors and senescence in progeroid mice.

### 3.4 Combination of dapansutrile and lonafarnib improves inflammation, senescence and survival in Lmna^G609G^ ^/G609G^ mice

Recently, the farnesyltransferase inhibitor (FTI) lonafarnib has been approved by the FDA for the treatment of patients with HGPS. Although, lonafarnib unequivocally improves the condition of HGPS patients (Lonafarnib, 2021), its use is not without unwanted side effects, including the upregulating the expression of proinflammatory cytokines in HGPS fibroblasts (Arnold et al., 2021). According to this we decided to evaluate the efficacy of dapansutrile versus lonafarnib and the combination of both drugs.

**FIGURE 3:**
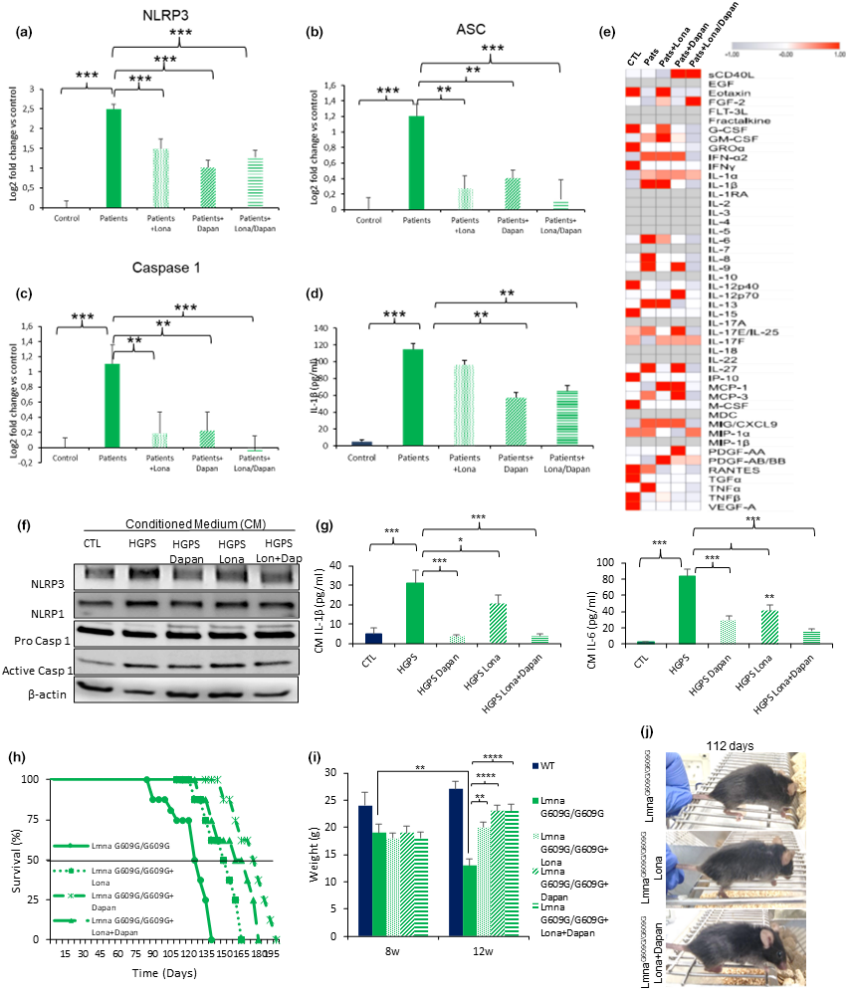
Dapansutrile works as a co‐treatment of lonafarnib in Lmna^G609G/G609G^ mice. (a–d) Effect of lonafarnib, dapansutrile, and lonafarnib combined with dapansutrile on mRNA levels of NLRP1, NLRP3, ASC and Caspase 1 determined by qPCR experiments in skin fibroblasts, *n* = 3 (controls) and three patients per group. (e) Heat map depicting expression of 48 human cytokines in the medium of skin fibroblasts with lonafarnib, dapansutrile and lonafarnib combined with dapansutrile, *n* = 3 (controls) and *n* = 3 (patients) per group. (f, g) Effect of culturing macrophages in conditioned medium (CM). CM was collected from control, HGPS, HGPS+lonafarnib, HGPS+dapansutrile and HGPS+lonafarnib combined with dapansutrile fibroblasts. Protein levels of NLRP1, NLRP3, and caspase 1 are shown in (f, Densitometry in Figure S9), and levels of IL1β and IL‐6 release in (g). (h) Kaplan–Meier graph showing a significant increase in the medium and maximum lifespan in untreated versus treated Lmna^G609G/G609G^ mice with the different treatments indicated. *N* = 8 per group. (i) Body weights of the indicated groups at 8 and 12 weeks after the indicated treatment. (j) Representative image of kyphosis of untreated and treated Lmna^G609G/G609G^ mice with lonafarnib and lonafarnib+dapansutrile. Data are shown as means ± SD. ****p < 0.0001, ***p < 0.001, **p < 0.01, *p < 0.005.

We analyzed the expression levels of several genes of the inflammasome complex, including NLRP3, NLRP1, caspase‐1, and ASC in HGPS fibroblasts in response to the aforementioned drugs, either individually or in combination. Our findings showed that the expression of NLRP3, ASC, and Caspase 1 from HGPS fibroblasts was reduced by lonafarnib and lona+dapan, being more significant in the effector caspases 1, however, no treatment altered NLRP1 expression levels (Figure 3a–c and Figure S8a). Interestingly, dapansutrile and the combination lona+dapan showed a more significant reduction of IL‐1β release compared with lonafarnib (Figure 3d). Furthermore, only the combination of both drugs was effective in reducing IL‐6 and IL‐18 levels (Figure S9). The treatments demonstrated a statistically significant dose‐dependent increase in the growth rates of HGPS fibroblasts treated with dapansutrile, and an improvement of the effect of lonafarnib when combined with dapansutrile, indicating a notable impact of the co‐treatment with both drugs (Figure S8b). Furthermore, the anti‐inflammatory effect was further corroborated by the results of the SASP analysis from HGPS fibroblasts. Again, Lona+Dapan showed a significant anti‐inflammatory effect compared with the independent treatments (Figure 3e).

We hypothesized that the NLRP3 inflammasome in HGPS could be regulated by the SASP and other senescence‐associated components. To test this hypothesis, we exposed THP‐1‐derived macrophages to conditioned medium (CM) from control and HGPS fibroblasts exposed to the different treatments studied. We found that CM from patient cells induced NLRP3, caspase 1 protein expression and IL‐1β and IL‐6 release. The response was attenuated in cells treated with dapansutrile and the combination (lona+dapan) (Figure 3f,g and Figure S10). These data strongly suggest that senescence in progeria fibroblasts promotes the inflammatory response dependent on the NLRP3 signalling pathway.

Finally, we explored the effects of the pharmacological inhibition of NLRP3 by dapansutrile in combination with lonafarnib in vivo. We studied four groups: (i) homozygous Lmna^G609G/G609G^ without any treatment, (ii) treated with lonafarnib, (iii) treated with dapansutrile and (iv) treated with lona+dapan. We found that treatment with lonafarnib resulted in a significantly extended survival of Lmna^G609G/G609G^ mice. The former was further improved by the combination of lonafarnib and dapansutrile. Surprisingly, dapansutrile alone extended survival more alone than in combination with lonafarnib (Figure 3h). Lonafarnib treatment resulted in an improvement in body weight, increasing from 14.7 ± 0.7 g to 18.6 ± 0.7 g (*p* < 0.001), with a more pronounced effect observed in the other two groups receiving dapansutrile (Figure 3i). According to the other scored phenotypes, we found that the percentage of animals with kyphosis was reduced in lonafarnib and lona+dapan treated mice (Figure 3j).

## 4 DISCUSSION

During the last years, there has been an intensive search for treatments for HGPS. Nevertheless, most of previous studies had insufficient preclinical data for translation into clinical trials in patients with HGPS. The most notable study focuses on the inhibitor of farnesyltransferase, known as lonafarnib, which was recently approved by the FDA (Lonafarnib, 2021). However, lonafarnib has several side effects including the formation of donut‐shaped nuclei and cell death observed in invitro experiments after a long‐term treatment. Furthermore, it has not known effect on inflammation that is a key component in HPGS (Arnold et al., 2021; Lee et al., 2016; Verstraeten et al., 2011). So, to improve the efficacy of lonafarnib treatment in HGPS, compounds abrogating FTI‐negative cellular effects and ameliorating cell functions not restored by FTI are urgently needed. One novel potential strategy is to reduce the downstream toxic effect of progerin at the cellular level. Recent studies have reported that chronic low‐grade inflammation may be a common etiology for various pathologies affecting patients with HGPS, in which the NLRP3 was also recently implicated (González‐Dominguez et al., 2021; Liu et al., 2019; Squarzoni et al., 2021). In fact, progerin has been shown to induce inflammation and activate NLRP3‐inflammasome and the downstream cytokine IL‐1β (Bidault et al., 2020; González‐Dominguez et al., 2021). This and our previous work showed a significant reduction of progerin levels after NLRP3 inhibition which would be associated to a reduction of cellular stress with inflammatory consequences. Accordingly, any strategy reducing inflammation could elicit a synergistic effect with lonafarnib.

Only a bunch of anti‐inflammatory strategies for the treatment of HGPS have been explored (Lai & Wong, 2020). However, most of the drugs trialed to treat HGPS, such as metformin, resveratrol, rapamycin, quercetin, or spermidine, only indirectly inhibit inflammatory pathways, including the NLRP3 inflammasome (Cordero et al., 2018). In the current study, we provide evidence demonstrating that inhibition of the NLRP3‐inflammasome complex in both human cells and in a mouse model of HGPS with dapansutrile, a safe NLRP3 inhibitor in human, improved cell survival and morphology, reduced inflammation and progerin levels and decreased senescence and SASP markers. Further dapansutrile treatment resulted in extended longevity, reduced kyphosis and, increased body weight gains in HGPS mice. Therefore, this HGPS treatment strategy, focused on the inhibition of NLRP3 inflammasome complex, could constitute an alternative therapy to slow down disease progression in patients with progeria. Previously, we showed that other inhibitor of NLRP3, MCC950 improved the longevity in Zmpste24^−/−^ mice. However, MCC950 has been associated with hepatic toxicity (Coss, 2020). Now, our findings reveal that other NLRP3 inhibitor, dapansutrile, improves both the lifespan and health span of Lmna^G609G/G609G^ mice, suggesting a potential synergistic effect when used in combination with lonafarnib. According to this, the second part of our study was to evaluate the efficiency of the combination of both treatments. Interestingly, dapansutrile improved the effect of lonafarnib on inflammation, cell growth, senescence and SASP. Additionally, dapansutrile augmented the survival benefits associated with lonafarnib in Lmna^G609G/G609G^ mice, improved the body weight and reduced the kyphosis apparition.

The efficacy of dapansutrile has been evaluated in animal models of various inflammatory diseases with very promising results. This includes bone diseases like acute gout or osteoarthritis, cardiovascular conditions such as myocardial ischemia/reperfusion injury or infarction, and neurodegenerative diseases, including multiple sclerosis and Alzheimer's disease (Aliaga et al., 2021; Cao et al., 2022; Lonnemann et al., 2020; Sánchez‐Fernández et al., 2019; Tang et al., 2022). This is important since bone and cardiovascular defects are the principal clinical manifestation in HGPS (Cabral et al., 2023; Lai & Wong, 2020). In this sense, Dapansutrile is being evaluated in clinical trials, showing promising beneficial effects in various inflammatory diseases such as cardiovascular disease, gout (Jansen et al., 2019; Klück et al., 2020; Wohlford et al., 2020), Diabetes mellitus (NCT06047262), COVID (NCT04540120) and osteoarthritis (NCT02104050). These trials collectively support dapansutrile as a new and safe anti‐inflammatory drug, highlighting its potential as a co‐treatment in degenerative disease such HGPS. Lonafarnid has shown clear benefits in the treatment of HGPS, yet its use is associated with side effects linked to inflammatory pathways and cell death mediated by the NLRP3 inflammasome, a process known as pyroptosis (Coss, 2020). In fact, lonafarnib has been shown to cause increased frequency of cytosolic DNA fragment formation, which activates the cGAS‐STING‐STAT1 signalling axis and upregulates the expression of proinflammatory cytokines (Arnold et al., 2021). For these reason, anti‐inflammatory strategies have been tested and proposed as treatment and co‐treatment for HGPS. The modulation of complexes inflammatory pathways shows an interesting target. Indeed, our findings are in consonance with previous data about IL‐6 and JAK/STAT1 pathways inhibition. Specifically, specific inhibition of Il‐6 by tocilizumab demonstrated to improve inflammation and phenotype of both patient fibroblasts and mouse model of HGPS reducing the accumulation of progerin, and rescuesing the nuclear envelope and chromatin abnormalities (Squarzoni et al., 2021). Furthermore, recent studies showed that aberrant activation of JAK–STAT signalling increase of IL6 and IL8 in HGPS cells and animal models which was reduced by Baricitinib treatment, a specific JAK1/2‐STAT1/3 inhibitor (Arnold et al., 2021; Hartinger et al., 2023). These finding support our results about the importance of the inflammation inhibition in HGPS. Besides, a direct relation has been shown between inflammation and progerin levels (Arnold et al., 2021; Bidault et al., 2020; Hartinger et al., 2023; Squarzoni et al., 2021) which also support our findings about the reduction of progerin after NLRP3 inhibition.

In conclusion, our study provided evidence that combining two drugs well‐tested and tolerated in humans (lonafarnib with dapansutrile), exhibited positive synergistic effects on human HGPS fibroblast homeostasis and demonstrated beneficial effect in an in vivo model of HGPS. The efficacy of this combination therapy, involving lonafarnib and dapansutrile, requires further evaluation to determine its potential as a therapeutic strategy for children with HGPS and possibly other age‐related conditions. However, the preliminary findings appear promising.

## AUTHOR CONTRIBUTIONS

M.D.C., and A.S., designed the study. I.M.Z, J.M.S.R., D.B.R., J.L.P., M.S.M., and M.M.N.A., performed the experiments. I.G.C., and J.M.B.A., performed chemical drug availability for in vitro and in vivo experiments. M.D.C., A.S., I.G.C., and J.M.B.A., discussed and analyzed the data. MD.C., and A.S., drafted the manuscript. All the authors revised the manuscript and approved the final version.

## FUNDING INFORMATION

MDC is supported by PI21/01656 grant from Instituto de Salud Carlos III, Spain and PRF 2021–78 from Progeria Research Foundation. AS is supported by Wellcome Senior Research Fellowship (212241/A/18/Z) & BBSRC grants (BB/R008167/1 & BB/W006774/1). Funding for open access publishing: Universidad Pablo de Olavide/CBUA.

## CONFLICT OF INTEREST STATEMENT

The authors declare that the research was conducted in the absence of any commercial or financial relationships that could be construed as a potential competing interest.

## Supporting information


**Appendix S1:** Supporting Information.

## Data Availability

The data that support the findings of this study are available from the corresponding author upon reasonable request.
