# Supplementary material for: The NLRP3 inhibitor Dapansutrile improves the therapeutic action of lonafarnib on progeroid mice
Source: Aging Cell. 2024 Aug 27;23(9):e14272. doi: 10.1111/acel.14272 (PMC11488313; doi:10.1111/acel.14272)
Supplement: Supplementary file 1 — Appendix S1: Supporting Information. [file ACEL-23-e14272-s001.pdf]

Supplementary data

The NLRP3 inhibitor Dapansutrile improves the therapeutic action of lonafarnib on progeroid mice

Inés Muela-Zarzuela[1], Juan Miguel Suarez-Rivero[1], Daniel Boy-Ruiz[1], Juan López-Pérez[2,3], Marta Sotelo-Montoro[1], Maria del Mar Navarrete-Alonso[1], Isidro G. Collado[4], José Manuel Botubol-Ares[4], Alberto Sanz[5], Mario D. Cordero [1]

[1] Department of Molecular Biology and Biochemical Engineering, Universidad Pablo de Olavide, 41013 Seville, Spain.

[2] Department of Immunology, Puerta del Mar Hospital, 11009 Cádiz, Spain.

[3] Instituto de Investigación e Innovación Biomédica de Cádiz, INiBICA, Hospital Universitario Puerta del Mar, Cádiz, Spain.

[4] Departamento de Química Orgánica, Facultad de Ciencias, Campus Universitario Río San Pedro s/n, Torre Sur, 4a planta, University of Cádiz, Puerto Real, 11510 Cádiz, Spain.

[5] School of Molecular Biosciences, College of Medical, Veterinary and Life Sciences, University of Glasgow, G12 8QQ, Glasgow, United Kingdom.

Running Title: Dapansutrile improves progeria phenotypes

Corresponding Author:

Department of Molecular Biology and Biochemical Engineering, Universidad Pablo de Olavide, 41013 Seville, Spain. Email: mdcormor1@upo.es.

Supplementary Table 1. Primers sequence for qPCR.

**Gene**	**Forward 5’-3’**	**Reverse 5’-3’**
NLRP1	TCTCTGCCTGCCTGATACCC	ACCTCCATGCCACTCGTCTT
NLRP3	CGTCTGGGTGAGAGTGTGAG	TCATCGGGGTCAAACAGCAA
ASC	AGCCAGGCCTGCACTTTATA	CATCTTGCTTGGGTTGGTGG
Caspase 1	GCCTGTTCCTGTGATGTGGAG	TGCCCACAGACATTCATACAGTTTC

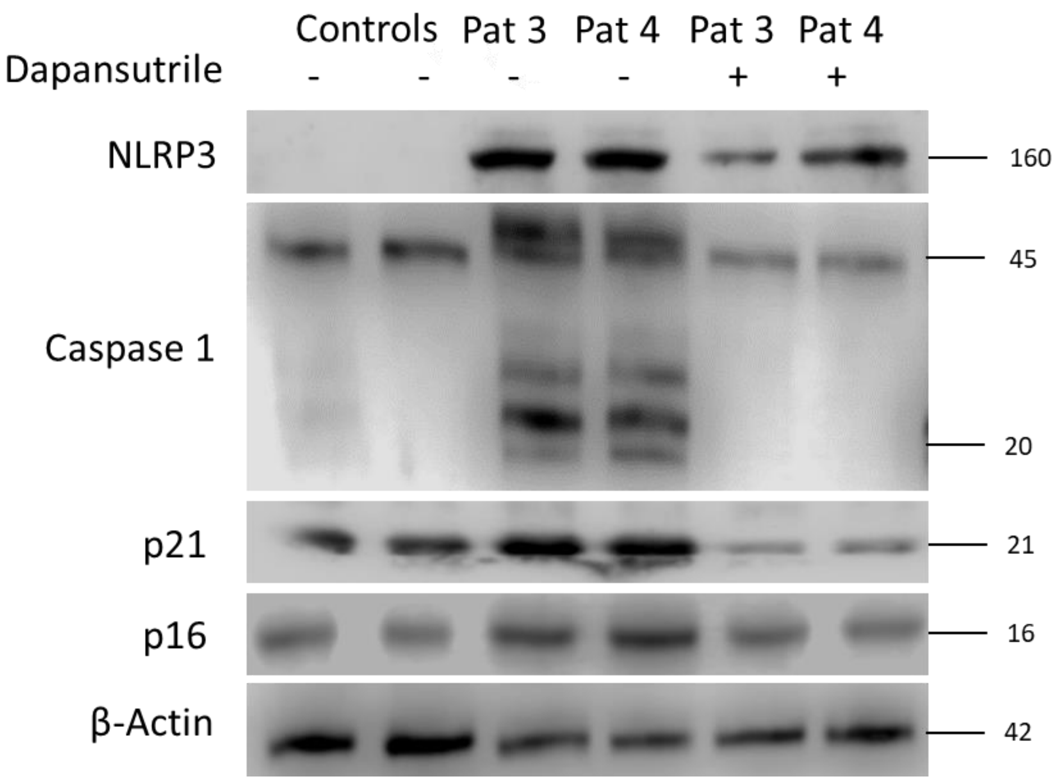

Supplementary Figure 1. Effect of dapansutrile in inflammasome and senescence protein expression. Western blot analysis showing representative blots of NLRP3, caspase 1, p16, p21 and β-actin levels in skin fibroblasts from patients with HGPS, n = 2 (controls) and n = 2 (patients), different from the Figure 1.

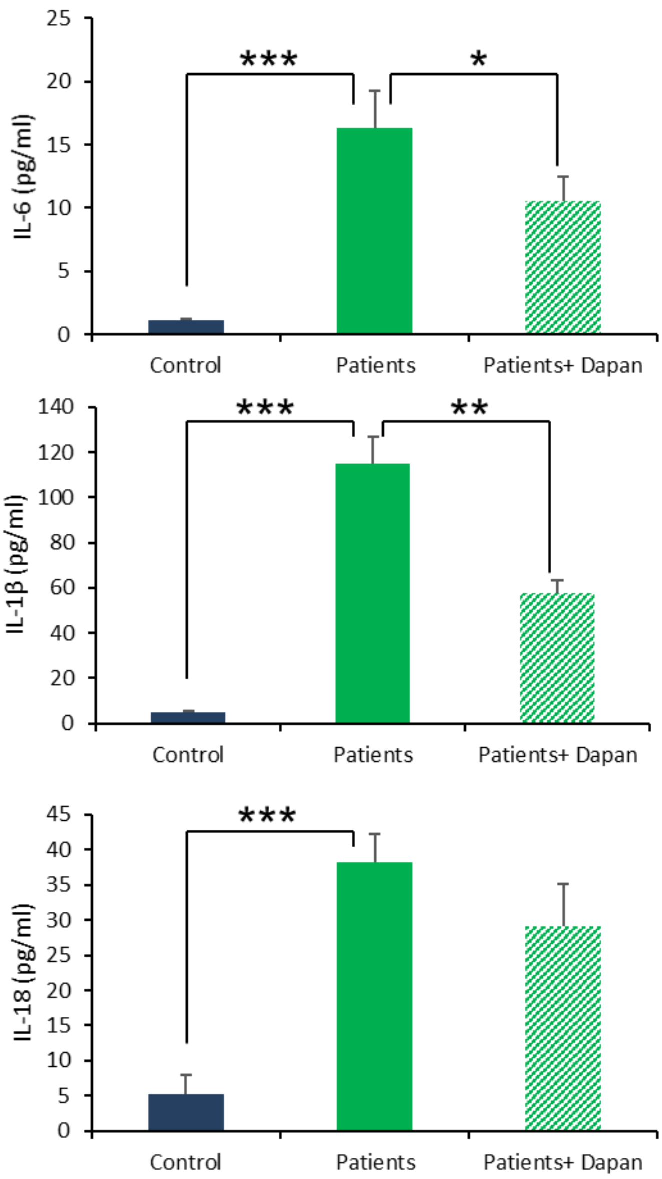

Supplementary Figure 2. Effect of the Dapansutrile on cytokine releases. IL-6, IL-1 and IL-18 were determined by ELISA in overnatant from control fibroblasts and HGPS patients cells. Data are shown as means ± SD. ***P < 0.001, **P < 0.005, *P < 0.05.

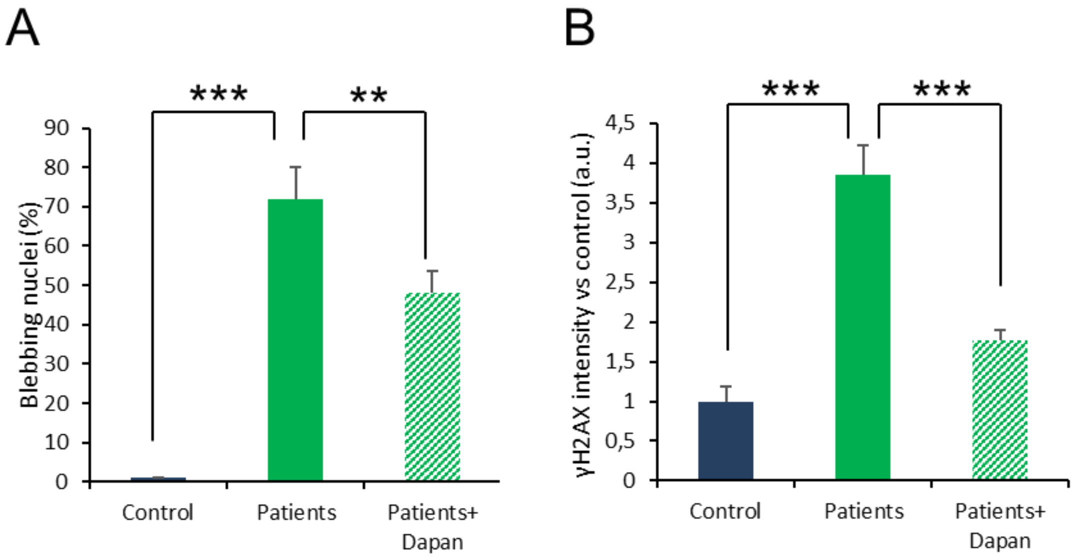

Supplementary Figure 3. Effect of Dapansutrile on nuclear dysfunction. A. Percentage of nucleus with abnormal morphology, associated to HGPS phenotype. B. Quantitation of nuclear γH2AX staining in healthy and HGPS fibroblasts after dapansutrile treatment. Data are shown as means ± SD. ***P < 0.001, **P < 0.005.

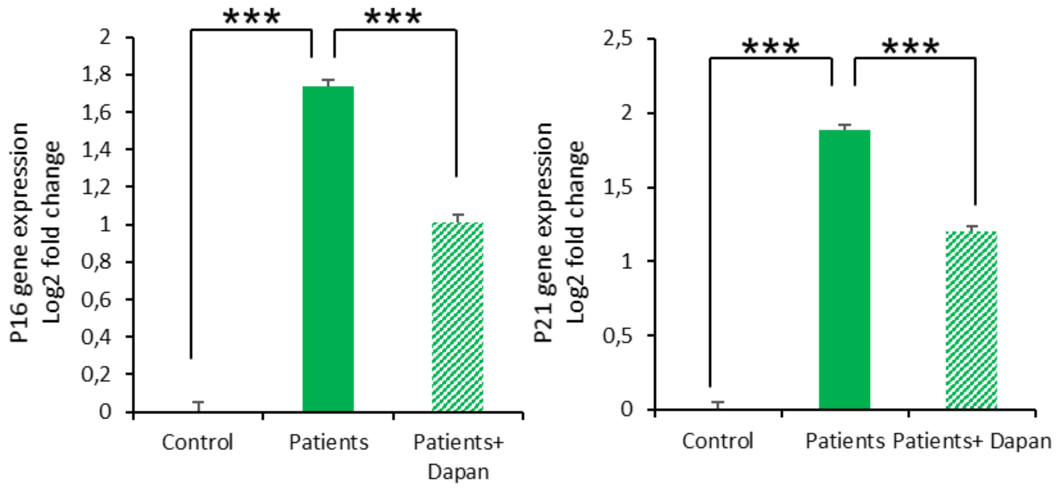

Supplementary Figure 4. Dapansutrile can reduce senescence expression markers. Effect of Dapansutrile on mRNA expression of p16 and p21 determined by qPCR experiments in skin fibroblasts, n = 3 (controls) and n = 8 (patients). Data are shown as means ± SD. ***$P < 0.001$.

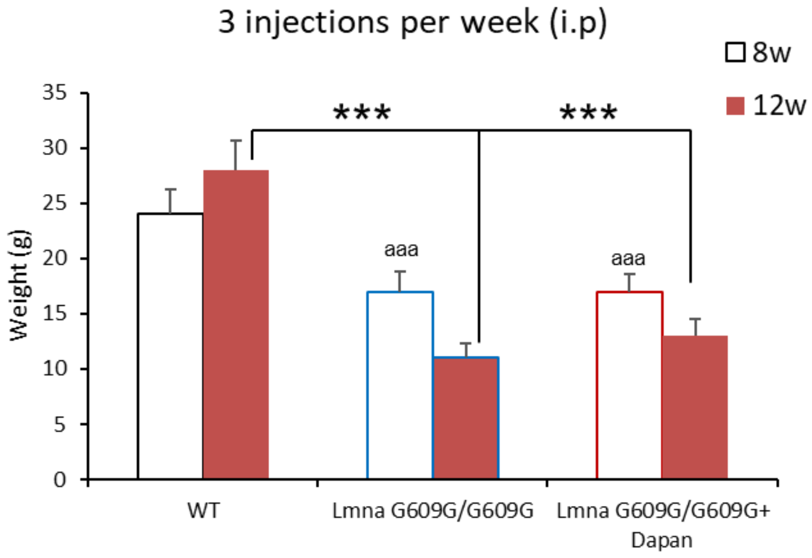

Supplementary Figure 5. Body weights of the indicated groups at 8 and 12 weeks after i.p. treatments. N= 6 per group. Data are shown as means ± SD. ***P < 0.001. ^{aaa}P < 0.001 Lmna $^{G609G/G609G}$ vs wt mice.

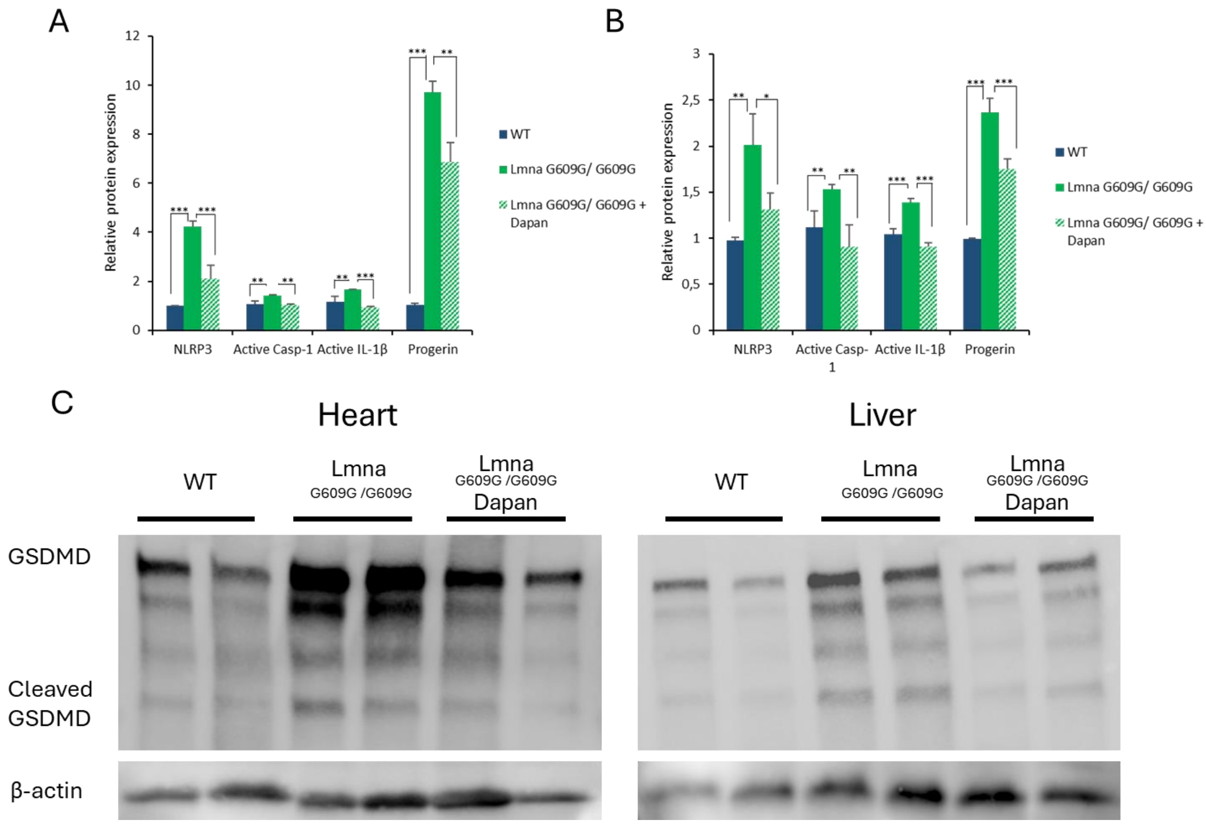

Supplementary Figure 6. Densitometric analisis of NLRP3, Active caspase 1, active IL-1β and progerin protein expression in heart (A) and liver (B) from WT, HGPS mice and HGPS mice treated with dapansutrile. C. Western blot analysis showing representative blots of GSDMD and β-actin levels in N= 6 per group. Data are shown as means ± SD. ***P < 0.001, **P < 0.01, *P < 0.05.

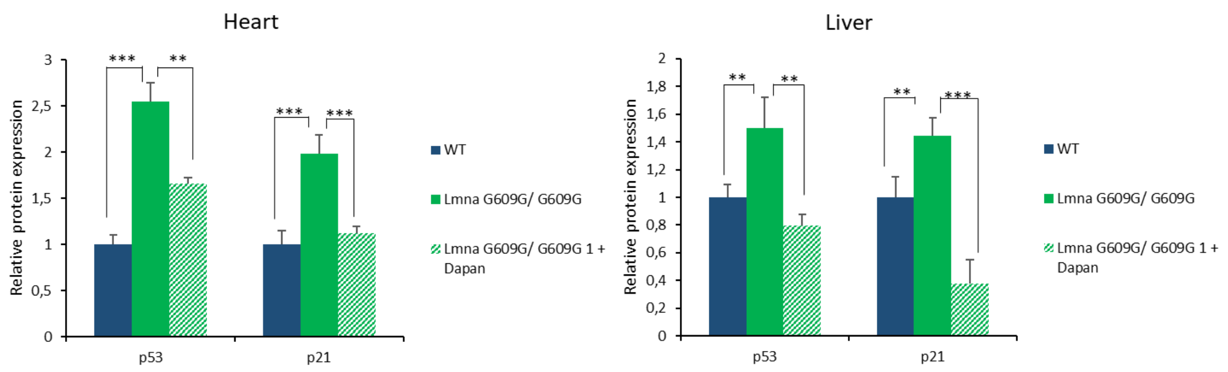

Supplementary Figure 7. Densitometric analisis of p53 and p21 protein expression in heart (left) and liver (right) from WT, HGPS mice and HGPS mice treated with dapansutrile. N= 6 per group. Data are shown as means ± SD. ***P < 0.001, **P < 0.01, *P < 0.05.

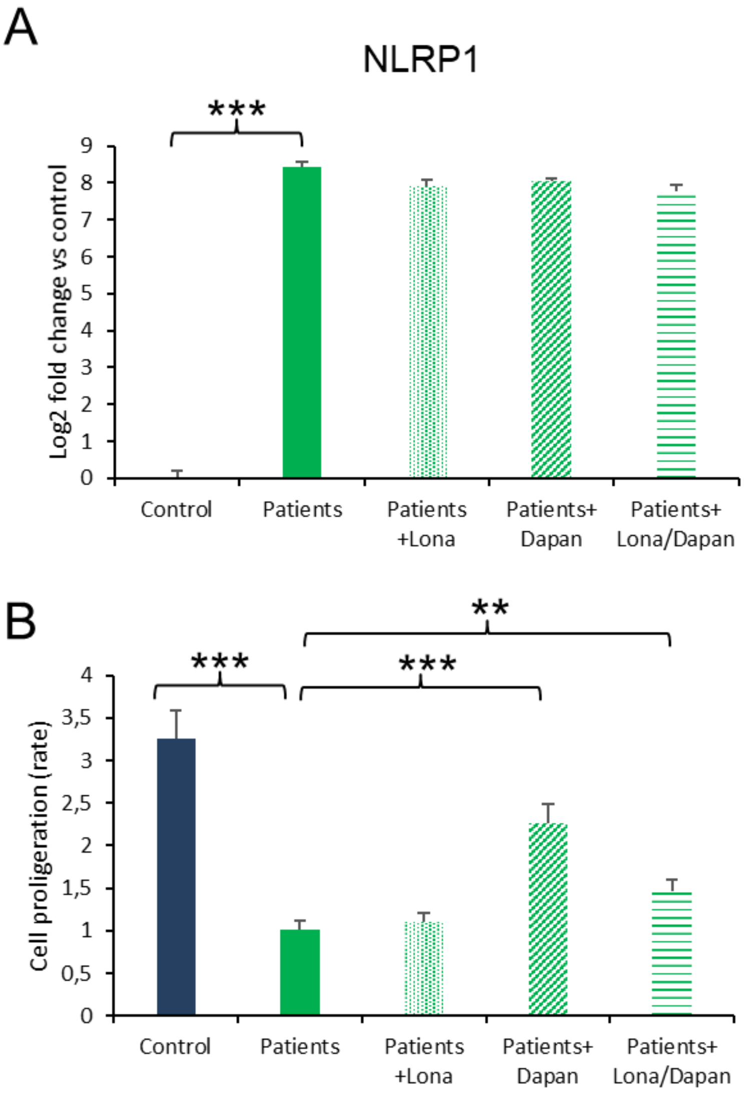

Supplementary Figure 8. Dapansutrile can improve cell proliferation but not reduce NLRP1 expression. A. Effect of Dapansutrile, lonafanib and the combination on mRNA expression of NLRP1 determined by qPCR experiments in skin fibroblasts. B. Cell growth with Dapansutrile, lonafanib and the combination determined in healthy and HGPS fibroblasts from 8 patients. n = 3 (controls) and n = 8 (patients). Data are shown as means ± SD. ***$P < 0.001$, **$P < 0.005$.

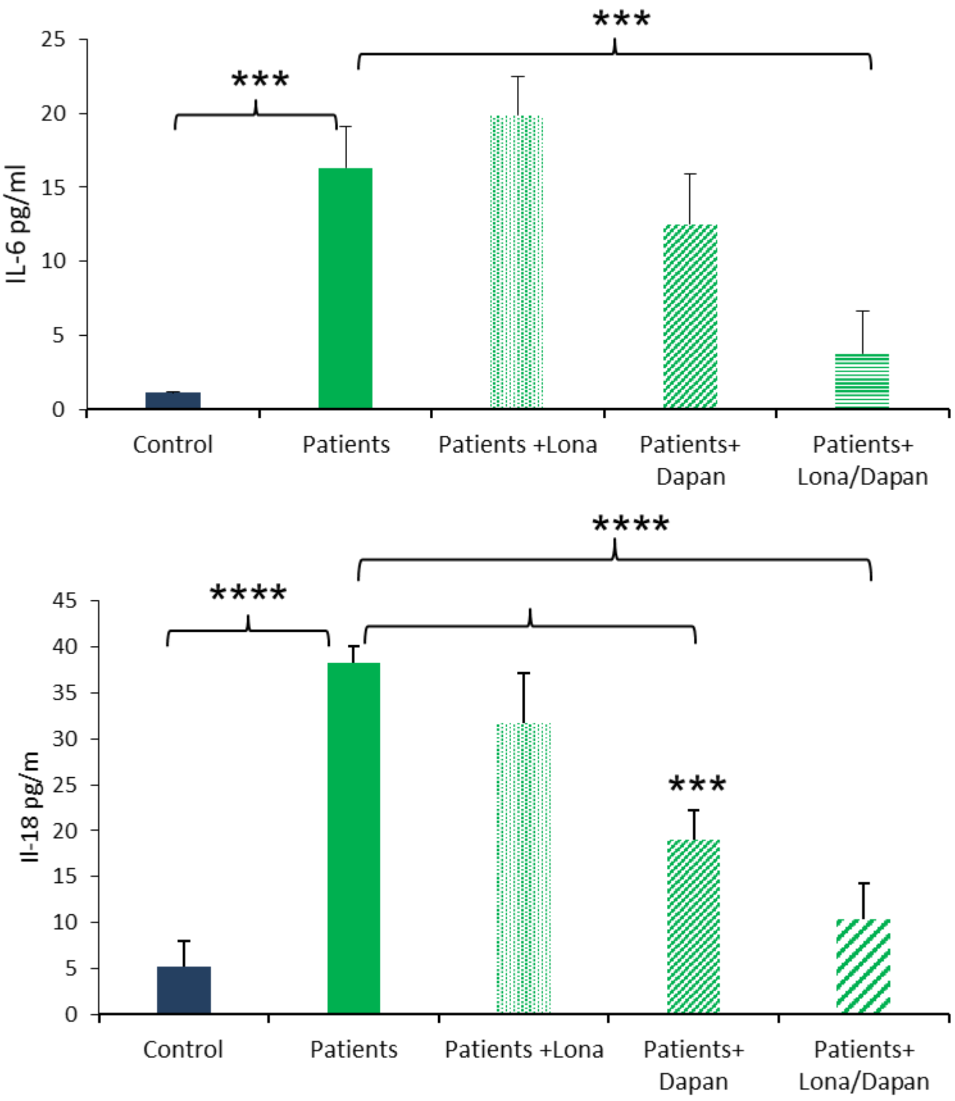

Supplementary Figure 9. Effect of the Dapansutrile, lonafarnib and the combination on cytokine releases. IL-6, IL-1 and IL-18 were determined by ELISA in overnatant from control fibroblasts and HGPS patients cells. Data are shown as means ± SD. ****P < 0.0001, ***P < 0.001.

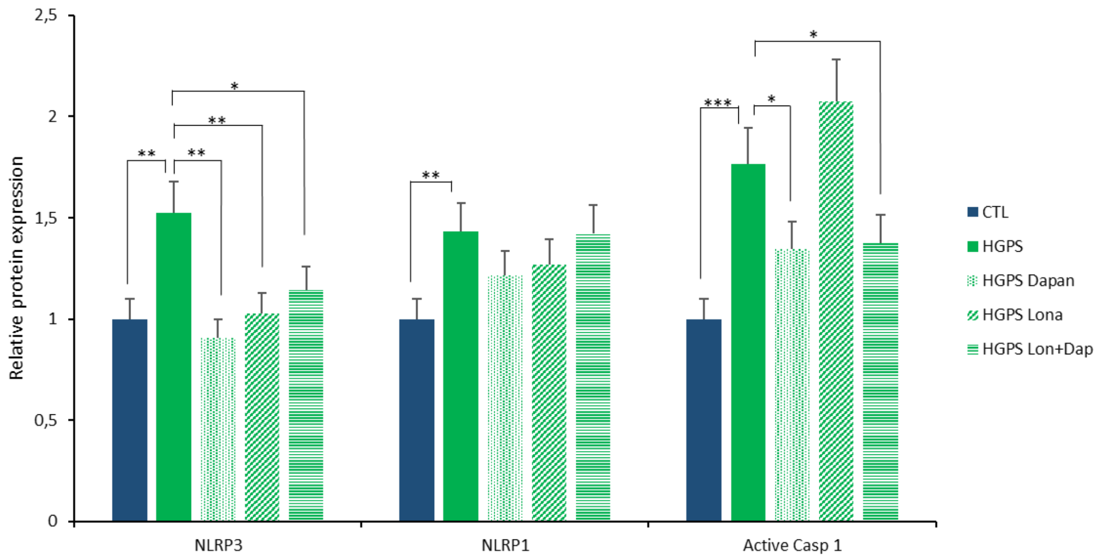

Supplementary Figure 10. Densitometric analisis of NLRP3, NLRP1 and Active caspase 1 protein expression in cultured fibroblasts with conditional media. Data are shown as means ± SD. ***P < 0.001, **P < 0.01, *P < 0.05.
